# Iodine Status in Schoolchildren and Pregnant Women of Lazio, a Central Region of Italy

**DOI:** 10.3390/nu11071647

**Published:** 2019-07-18

**Authors:** Enke Baldini, Camilla Virili, Eleonora D’Armiento, Marco Centanni, Salvatore Ulisse

**Affiliations:** 1 Department of Surgical Sciences, “Sapienza” University of Rome, 00161 Rome, Italy; 2 Department of Medico-Surgical Sciences and Biotechnologies, “Sapienza” University of Rome, 04100 Latina, Italy; 3 Department of Internal Medicine and Medical Specialties, “Sapienza” University of Rome, 00161 Rome, Italy

**Keywords:** iodine deficiency, schoolchildren, pregnancy, iodine prophylaxis, iodine deficiency disorders, goiter, hypothyroidism

## Abstract

The inhabitants of Lazio, similarly to those of other Italian regions, have been historically exposed to the detrimental effects of an inadequate intake of iodine. The latter is a micronutrient essential for the biosynthesis of thyroid hormones (TH). Iodine deficiency is responsible for a number of adverse effects on human health known as iodine deficiency disorders (IDD), the most common of which worldwide are goiter and hypothyroidism. In order to reduce IDD, a national salt iodination program was started in Italy in 2005. In this article we reviewed the available data regarding iodine intake in the Lazio population before and after the introduction of the national salt iodination program, in order to evaluate its efficacy and the eventual problem(s) limiting its success. On the whole, the information acquired indicates that, following the introduction of the program, the dietary iodine intake in the Lazio population is improved. There is, however, still much work ahead to ameliorate the iodine prophylaxis in this region. In fact, although a generally adequate iodine intake in school-age children has been observed, there are still areas where a mild iodine insufficiency is present. Moreover, two independent epidemiological surveys on pregnant women evidenced a low urinary iodine concentration with respect to the reference range conceived by the World Health Organization. These findings demonstrate the need for greater attention to the iodine prophylaxis by health care providers (i.e., obstetricians, gynecologists, pediatricians, etc.), and the implementation of effective advertising campaigns aimed at increasing the knowledge and awareness of the favorable effects of iodine supplementation on population health.

## 1. Introduction

Iodine is an indispensable micronutrient required by the thyroid gland for the appropriate synthesis of the thyroid hormones (TH), i.e., triiodothyronine (T_3_) and its prohormone thyroxine (T_4_) [1]. By modulating key cellular processes (i.e., proliferation, differentiation, apoptosis, and metabolism), TH affect multiple body tasks from the early stages of prenatal life, when maternal thyroxinemia plays a fundamental role in neural growth and differentiation, to adulthood, in which they regulate metabolism, thermogenesis, feeding, memory/learning abilities, and cardiovascular and reproductive functions [2,3,4,5]. To guarantee an appropriate TH biosynthesis, the daily dietary iodine intake recommended by the World Health Organization (WHO), the United Nations Children’s Emergency Fund (UNICEF), and the International Council for the Control of Iodine Deficiency Disorders (ICCIDD) is 90 μg for preschool children (0 to 59 months), 120 μg for schoolchildren (6 to 12 years), 150 μg for adolescents (above 12 years) and adults, 250 μg for pregnant and lactating women [6]. Failure to meet these requirements is held responsible for a number of adverse effects on human health known as iodine deficiency disorders (IDD) [7]. These affect almost 1.9 billion people worldwide and constitute a major public health issue in different countries, including Italy [6,8,9]. IDD may occur at all ages, from the early stages of fetal life to adulthood. Of particular relevance are the detrimental effects of an insufficient maternal intake of iodine on development and maturation of the fetal brain, which represents a foremost preventable cause of mental defects [10]. Further adverse effects include abortion, stillbirth, impairment of cognitive functions, delayed growth and puberty, hypothyroidism, goiter, and infertility [11,12,13,14,15,16,17]. 

The epidemiological criteria established by the WHO to evaluate the prevalence and severity of iodine deficiency in a specific population refer to the median urinary iodine concentration (UIC) in morning spot urine samples, along with the presence of goiter [6]. Assuming a daily diuresis of 1.5 liters, a given land area is considered iodine sufficient when the median UIC of the population is comprised between 100 and 199 μg/L, and goiter prevalence in school-age children (≥6 years) is below 5% [6]. In pregnant women, median UIC should be comprised between 150 and 250 μg/L to guarantee normal fetal development. Reported in Table 1 are the WHO reference values of UIC for classifying the iodine status in a population.

In the attempt to establish an efficient iodine prophylaxis and to eradicate IDD, the law n.55/2005, introducing a national salt iodination program (30 mg of potassium iodate per kilogram of salt), was promulgated in 2005 in Italy [9]. The rules laid down by this law make the sale of iodized salt compulsory and favor the silent prophylaxis. Specifically, the sale points of salt for direct consumption have to expose iodine enriched salt while ensuring the availability of non-iodized salt, which is provided only upon specific request of the consumer. In the public catering sector, such as bars and restaurants, and in workplace or community canteens, iodine-enriched salt should also be available to consumers. Furthermore, the law recommends the use of iodized salt in the food industries as an ingredient in preparation and food storage [9]. 

In the present manuscript, we will review the available information on iodine status in Lazio (a region of central Italy) before and after the introduction of the Italian law n.55/2005, along with the encountered problems hampering the actuation of the national iodine prophylaxis program. All papers analyzed have been obtained from PubMed. Additional data are from the National Observatory for the Monitoring of Iodoprophylaxis in Italy (OSNAMI) of the Italian National Institute of Health.

## 2. Iodine Status in the Lazio Region before the Introduction of the National Iodine Prophylaxis Program

The Italian population, including the inhabitants of Lazio, has historically been exposed to the negative effects of iodine food shortages [9,18,19,20]. 

**Figure 1:**
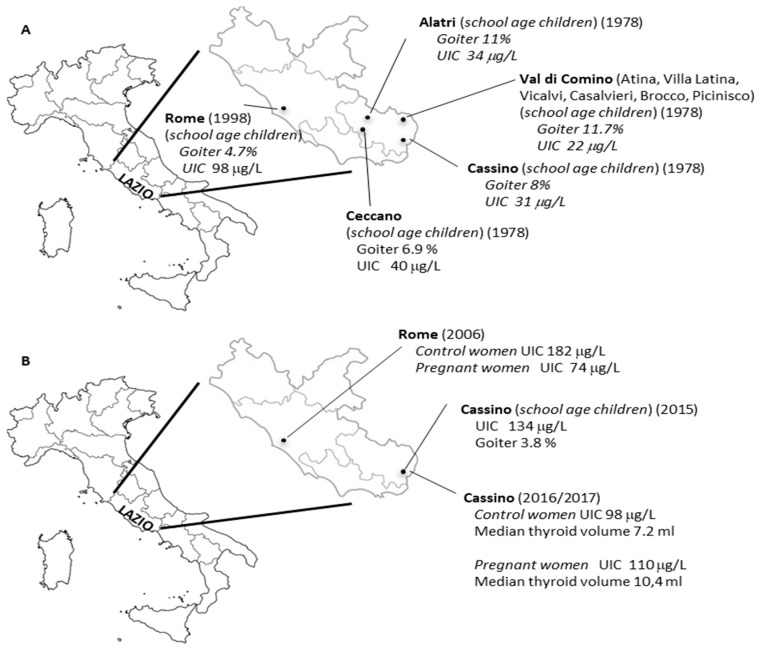
Urinary iodine concentrations (UIC) and goiter prevalence in the inhabitants of Lazio before (**A**) and after (**B**) the introduction of the national salt iodination program.

In the seventies of the last century, epidemiological studies carried out in about 5700 school-age children (6–13 years old) of southern Lazio documented the presence of iodine deficiency [21]. Reported mean UIC values varied from 22 µg per gram of creatinine (µg/g Cr) to 40 µg/g Cr, consistent with an iodine deficiency of moderate degree, as shown in Figure 1 and Table 1. In these children the prevalence of goiter, evaluated by palpation, varied from 6.9% to 11.7%, as shown in Figure 1. In a subsequent case study, performed in 1998 in the city of Rome, UIC and goiter prevalence were investigated in 1040 school-age children (6–14 years old) [22]. A median UIC value of 92 µg/L (mean UIC value of 98 µg/L), consistent with a mild iodine deficiency, as shown in Table 1, was still observed, as shown in Figure 1. On the other hand, goiter prevalence determined by ultrasound was 4.7% [22]. Hence, before the introduction of the nationwide iodine prophylaxis program, the data collected indicated a condition of mild to moderate iodine deficiency of children residing in Lazio. To the best of our knowledge, no studies were performed on iodine status in pregnant women before 2005.

## 3. Iodine Status in the Lazio Region after the Introduction of the National Iodine Prophylaxis Program

Following the introduction of the iodine prophylaxis program, three independent studies were performed in order to assess iodine status in both school-age children and pregnant women [23,24,25]. 

A first observational study was realized in 2006 in the city of Rome to evaluate iodine intake in pregnant women compared to non-pregnant age-matched ones [23]. The study enrolled 51 clinically healthy pregnant women in their first gestational trimester, and 100 clinically healthy non-pregnant women [23]. The median UIC value observed in control women was 182 μg/L, suggestive of an adequate iodine intake, as shown in Figure 1. However, pregnant women showed poor iodine consumption, attested by a median UIC value of 74 μg/L. In particular, the UIC was found below the normal range only in 4% of control women but in 92% of pregnant women. These observations pointed out that, despite the iodine sufficiency recognized in control women, the majority of pregnant women and their fetuses were exposed to detrimental consequences of iodine deficiency. 

After that, a new survey was performed in 2015 in the city of Cassino, located in the south of Lazio, whose residents were previously found to have a moderate iodine deficiency, as shown in Figure 1B [24]. In this study, UIC and thyroid volume measured by ultrasonography were evaluated in 234 school-age children (13–14 years old). At the same time an inquiry was conducted to estimate the percentage of iodized salt sold in the preceding year by the major local retailers. The latter showed only 42% of all salt sold in 2014 in this city was iodized. Despite that, a median UIC of 134 μg/L was observed in the school children under examination, suggesting an adequate iodine intake. This result was also corroborated by the low prevalence of goiter, encountered only in 3.8% of subjects. However, when children were grouped based on the regular consumption of iodized salt or milk or both, those referring no consumption of either iodized salt or milk had a median UIC value (96.4 μg/L), compatible with a mild iodine deficiency. On the other hand, optimal median UIC values were found in school children regularly taking either iodized salt (132.1 μg/L) or milk (131 μg/L) or both (147.9 μg/L). Such evidence strengthens the importance of eating iodized salt and iodine-rich food to achieve the right amount of iodine in the body for thyroid function [24]. 

A further study was aimed to analyze iodine intake in pregnant women from the same area of Cassino [25]. Study participants were enrolled in the period from January 2016 to April 2017, for a total of 96 pregnant women and 79 age-matched non-pregnant women. In the control group, median UIC was nearby 98 µg/L, consistent with a mild iodine deficiency, while pregnant women had a median UIC of about 110 µg/L, below the lower value (150 µg/L) recommended by the WHO for categorizing iodine adequacy in a pregnant population. In agreement with this finding, a significantly increased thyroid volume was recorded in pregnant women compared to non-pregnant ones, as shown in Figure 1 [25]. In this study the effects of iodized salt and/or milk consumption on UIC levels of both control and pregnant women, considered as a whole, were also examined. The analysis showed an increasing trend of UIC from women not using either iodized salt or milk (median UIC 79.8 µg/L) compared to those using iodized salt (median UIC 94 µg/L) or milk (median UIC 112 µg/L) or both (median UIC 118 µg/L) [25]. Thus, once again the data obtained highlight the need to implement the national salt iodination program, as well as the importance of inserting initiatives targeted at control of iodine prophylaxis and prevention of IDD in regional health plans. It has to be mentioned, however, that the aforementioned studies rely on a limited number of subjects analyzed and should be corroborated on larger case studies. 

## 4. Encountered Problems Hampering the Actuation of the National Iodine Prophylaxis Program 

As reported above, the available epidemiological data demonstrated that, following the introduction of the national salt iodination program, the iodine intake in the inhabitants of Lazio is somewhat improved. However, prevention measures are still needed to fully avoid the risks of IDD in this region. In particular, there are three main lines of action that should be pursued. 

The first is devising strategies to increment the amount of iodized salt sold by retailers. In fact, due to the lack of penalties related to non-compliance with the law n.55/2005, vendors are not discouraged from exhibiting non-iodized salt on store shelves, and they sell both iodized salt and or even just non-iodized salt [24]. This, at least in part, may explain the low percentage of iodized salt sold (42%) in the city of Cassino, and why 45% of the school children and 50% of women of child-bearing age do not use iodized salt. Moreover, the latest data provided by OSNAMI indicated that the percentage of iodized salt used in collective catering is still very low (about 24%), and that used by the food industry is even lower (3–8%) [26]. Thus, it is of great importance to encourage this manufacturing sector to make more extensive use of iodized salt. 

The second line of intervention should be to adequately inform the population on the beneficial effects on human health deriving from the consumption of iodized salt. This task could be accomplished by creating effective advertising campaigns able to reach every single citizen. In this context, an initiative of the Ministry of Health is taking place that aims to provide all students with comprehensive information about the beneficial effects of the use of iodized salt through meetings with medical staff organized by individual schools [27]. 

Finally, greater attention by the major health care providers of the National Health System is highly desirable, especially obstetricians, gynecologists, and pediatricians. Different surveys, in fact, noticed that obstetricians and midwives do not recommend iodine supplementation either to women planning pregnancy or during pregnancy or lactation [28,29]. To this regard, a position statement on the use of iodized salt in adulthood and children was signed by the Italian Ministry of Health, the National Institute of Health, the Italian Society of Endocrinology, the Italian Thyroid Association, the Medical Association of Endocrinologists, the Italian Society of Pediatric Endocrinology and Diabetology, the Italian Society of Pediatrics, the Italian Society of Gynecology and Obstetrics, the Italian Association of Consultative Gynecologists, the Italian Society of Human Nutrition, the Italian Society of Nutraceuticals, the Italian Association of Dietetics and Clinical Nutrition, the Italian Society for the Study of Food Behavior Disorders, the Italian Federation of Nutrition, and the National Federation of General Practitioners [30]. The joint implementation of all these actions should provide a consistent contribution toward the eradication of iodine deficiency in Italy. 

## 5. Conclusions

The available epidemiological data indicate that, following the introduction of the national salt iodination program, the iodine intake of the inhabitants of Lazio has only partially improved. In fact, although a generally adequate iodine intake in school-age children has been observed, pregnant women still show an iodine deficiency. Thus, it is necessary to encourage compliance with the law in order to reach optimal iodine nutrition and to completely eradicate the IDD in this region. The situation recorded in Lazio corroborates the recent Krakow Declaration on Iodine reporting an increased concern about the fading commitment of policymakers to address iodine deficiency in Europe and the poor attention of policymakers, opinion leaders, and the public toward the resolution of IDD [31]. Thus, it has become of primary importance to join forces with policymakers, public health officials, and scientists to guarantee that existing European strategies to prevent IDD are fulfilled and implemented.

## Figures and Tables

**Table 1:** Median urinary iodine concentrations (UIC) and iodine status in school-age children and pregnant women according to the World Health Organization (see reference [6]).

School-Age Children		Pregnant Women	
UIC (µg/L)	Iodine Status	UIC (µg/L)	Iodine Status
<20	Severe iodine deficiency	<150	Insufficient
20–49	Moderate iodine deficiency	150–249	Adequate
50–99	Mild iodine deficiency	250–499	Above requirements
100–199	Adequate iodine nutrition	≥500	Excessive
200–299	More than adequate		
≥300	Excessive		
